# OsRAD51 Plays a Vital Role in Promoting Homologous Recombination in Rice Meiosis

**DOI:** 10.3390/ijms23179906

**Published:** 2022-08-31

**Authors:** Xiaofei Liu, Yiwei Cao, Guijie Du, Chao Zhang, Meng Xu, Zhukuan Cheng, Yi Shen, Hengxiu Yu

**Affiliations:** 1 College of Life Science, Henan Normal University, Xinxiang 453007, China; 2 Jiangsu Key Laboratory of Crop Genomics and Molecular Breeding/Key Laboratory of Plant Functional Genomics of the Ministry of Education, Jiangsu Co-Innovation Center for Modern Production Technology of Grain Crops, College of Agriculture, Yangzhou University, Yangzhou 225009, China; 3 State Key Laboratory of Plant Genomics, Institute of Genetics and Developmental Biology, Chinese Academy of Sciences, Beijing 100101, China

**Keywords:** OsRAD51, OsDMC1, DSB repair, homologous recombination, meiosis

## Abstract

Meiotic recombination plays a pivotal role in achieving accurate chromosomal segregation and increasing genetic diversity. In the homologous recombination pathway, the detailed mechanisms of how OsRAD51 and OsDMC1 work in rice meiosis remain to be explored. Here, we obtained different types of mutants for *Osrad51a1*, *Osrad51a2*, *Osdmc1a*, and *Osdmc1b* through CRISPR/Cas9. Both *Osrad51a1* and *Osrad51a2* exhibited normal vegetative growth and fertility. *Osrad51* (*Osrad51a1 Osrad51a2*) mutant plants show normal vegetative growth but exhibit complete sterility, indicating that *OsRAD51A1* and *OsRAD51A2* are functionally redundant in rice fertility. In contrast to the wild type, *Osrad51* chromosomes are not paired perfectly at pachytene and synaptonemal complex (SC) formation is deficient. Moreover, univalents and multivalent associations were observed at metaphase I, chromosome fragments presented at anaphase I, and crossover formation is basically suppressed in *Osrad51* pollen mother cells (PMCs). OsRAD51 foci emerge at leptotene and disappear from late pachytene and chromosome localization of OsRAD51 depends on the formation of double-strand breaks (DSBs). Most OsRAD51 foci can co-localize with OsDMC1 signals. OsRAD51 is essential for the loading of OsDMC1 onto chromosomes, and vice versa. In addition, both OsRAD51 and OsDMC1 can interact with OsFIGL1 and OsBRCA2, two important components in rice meiosis. Moreover, the *Osrad51 Osdmc1* (*Osrad51a1 Osrad51a2 Osdmc1a Osdmc1b*) quadruple mutant PMCs exhibited similar defective phenotypes as *Osrad51* in homologous pairing, synapsis, and DSB repair. Taken together, our results suggest that the recombinases DMC1 and RAD51 may functionally depend on each other and play important roles in meiotic recombination during meiosis in rice.

## 1. Introduction

Meiosis is a specialized eukaryotic cell division process in which two successive rounds of nuclear segregation and one round of DNA replication lead to the formation of haploid gametes. After fertilization, the fusion of gametes restores the diploid chromosome number of the species. This process guarantees that organisms that undergo sexual replication maintain the same number of chromosomes. Moreover, the reshuffle of the genetic information in homologous chromosomes occurred during meiosis, which creates genetic diversity among offspring and is a primary source of genetic variation in eukaryotes [1,2].

Meiotic homologous recombination (HR) is an important biological event that occurs during meiosis. HR is initiated by the formation of double-strand breaks (DSBs), which are catalyzed by the action of conserved DNA transesterase SPO11 proteins [3,4]. In *Saccharomyces cerevisiae*, SPO11 proteins are covalently bonded to the 5′ end of a DSB and come off together with a short DNA oligonucleotide. This process is regulated by Sae2/Com1 and the Mre11/Rad50/Xrs2-Nbs1 (MRX/N) complex [5]. The MRX/N complex and Exo1 then cut the 5′ end to generate the 3′ end of the DSB in yeast [6,7]. After precise end-processing, the 3′ end is protected by the replication protein A complex (RPA), which is subsequently replaced when RAD51 and DMC1 are loaded, and form a nucleoprotein filament that can catalyze strand invasion in yeast and mammals [8,9]. DMC1 directs the formation of homologous joint molecules (JMs) between interhomolog chromosomes, and RAD51 can conduct JM formation between inter-sister chromosomes. The strand exchange activity of RAD51 and DMC1 has been demonstrated in vitro [10,11,12]. It has been shown in most eukaryotes RAD51 plays an essential role in both meiosis and mitosis, whereas DMC1 is a meiosis-specific protein [8,13].

RAD51 homologs have been identified in most of the eukaryotes examined to date and are extensively studied in human, mouse, and chicken. Meiotic DSBs accumulation and reduced formation of physical recombinants occur in the yeast *rad51* mutant [14]. Contrary to yeast, the *rad51* null mutation is lethal in the mouse and chicken [15]. RAD51 homologs have been identified in several plant species as well [16,17,18,19]. The *Atrad51* mutant shows partial synapsis during prophase I and massive chromosome fragmentation [18,20]. Immunolocalization assays reveal that RAD51 foci are located along the chromosome axes at zygotene in *Zea mays* (maize) and Arabidopsis [17,21]. In maize, RAD51 foci are associated with paired homologs, whereas either no or only a low level of RAD51 foci showed in pairing-defective mutants, demonstrating that RAD51 may play an important role in the homology search process in maize [19,22,23]. Recently, it has been reported that RAD51 functions as an interacting protein to restrain the SMC5/6 complex from inhibiting DMC1 [24]. These findings indicate that RAD51 is necessary for homologous chromosome pairing and meiotic recombination in a variety of organisms.

The function of RAD51 has been studied in some organisms, however, the functions of OsRAD51 during rice meiosis remain elusive. A previous study suggested that OsRAD51 is essential for somatic homologous recombination in DNA repair [25]. *OsRAD51A1* and *OsRAD51A2* are two conserved rice *RAD51* genes and share high sequence similarity. Whether they are functionally redundant in meiosis has not yet been determined. Moreover, the exact way that both OsRAD51 and OsDMC1 work in rice meiosis remains unclear. In this study, we constructed a CRISPR/Cas9 vector to edit the *OsRAD51A1*, *OsRAD51A2*, *OsDMC1A*, and *OsDMC1B* genes simultaneously. From this, we obtained *Osrad51a1 Osrad51a2* and *Osdmc1a Osdmc1b* mutants. The meiotic phenotype of the *Osrad51a1 Osrad51a2* mutant did not show full chromosomal pairing and synapsis at pachytene. At metaphase I, univalents and multivalents were observed, and a few fragmented chromosomes were present at anaphase I. Moreover, the meiotic phenotype of the quadruple mutant resembles the *Osrad51a1 Osrad51a2* mutant. Immunolocalization assays showed that most OsRAD51 foci can colocalize with OsDMC1 foci. Moreover, the localization of these two recombinases is interdependent. These results imply that OsRAD51 and OsDMC1 may function interdependently in rice meiotic recombination.

## 2. Results

### 2.1. Generation of Osrad51 and Osrad51 Osdmc1 Mutants by CRISPR/Cas9 Genome Editing

The Arabidopsis RAD51 amino acid sequence was used as a template in a BLAST search against the rice proteome [26]. We found two proteins that share a high level of similarity to AtRAD51 according to BLAST results. These two proteins, which are encoded by *LOC_Os11g40150* and *LOC_Os12g31370*, were named OsRAD51A1 and OsRAD51A2, respectively. OsRAD51A1 is predicted to consist of 339 amino acid residues. OsRAD51A2 has 341 amino acid residues, and the two putative proteins share 95% identity in the amino acid sequence. Both OsRAD51A1 and OsRAD51A2 contain an N-terminal helix-hairpin-helix DNA-binding motif class 1 domain (HhH1) and an ATPases associated with a variety of cellular activities domain (AAA) [27] (Supplementary Figure S1A). These two proteins were collectively named as OsRAD51 in this study. Multiple sequence alignment of OsRAD51 with orthologous proteins showed that it is highly evolutionarily conserved in eukaryotes (Supplementary Figure S1B). qRT-PCR assays revealed that *OsRAD51* expressed the highest level in young panicles of wild-type rice, but is significantly low in leaves and roots (Supplementary Figure S1C). 

**Figure 1:**
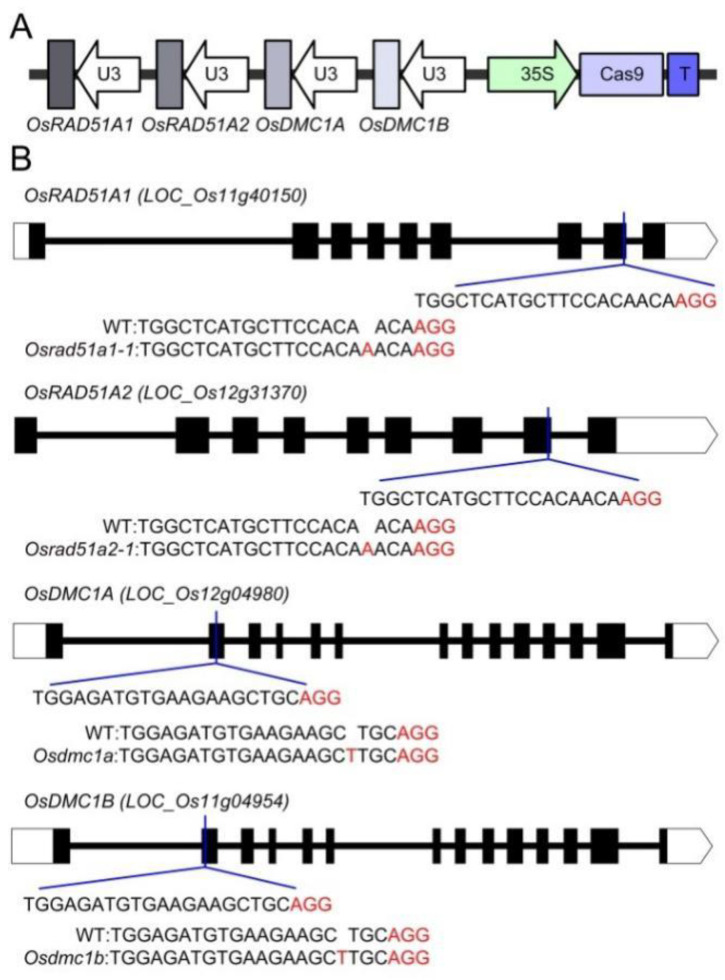
Targeted mutagenesis of *OsRAD51* and *OsDMC1* using the CRISPR/Cas9 genome editing system. (**A**). Strategy of vector construction for editing the rice *RAD51* and *DMC1* genes. (**B**). Regions in *OsRAD51* exon 8 and *OsDMC1* exon 2 were targeted by the CRISPR/Cas9 system. The positions of the sgRNAs are shown under the exons (black boxes) and the mutated sites in the mutants are shown beneath the genes aligned with the wild-type (WT) sequences. The PAM (protospacer adjacent motif) sites are shown in red.

Rice genomes contain two copies of *DMC1* (*OsDMC1A* and *OsDMC1B*) [28]. To study the relationships between both two recombinases in-depth, the quadruple mutant is needed. It would be extremely difficult to obtain the quadruple mutant using routine mutagenesis and crossbreeding. Therefore, we constructed a CRISPR/Cas9 vector to edit these four genes (Figure 1A). Through *Agrobacterium*-mediated transformation of the rice variety Yandao 8, seedlings of the first (T0) generation with specific mutations in *OsRAD51A1*, *OsRAD51A2*, *OsDMC1A*, and *OsDMC1B* were obtained. In the T1 generation, we obtained two *Osrad51a1 Osrad51a2* alleles and individually designated them *Osrad51-1* and *Osrad51-2* (the *Osrad51* mutants mentioned in this article are all *Osrad51-1*). One *Osdmc1a Osdmc1b* plant was designated *Osdmc1*, and one quadruple mutant was designated *Osrad51 Osdmc1*. We also isolated plants with single mutations in *Osrad51a1*, *Osrad51a2*, *Osdmc1a*, and *Osdmc1b*. To determine the genotype and heritability of every line, we sequenced the target genes to ascertain the genotypes in the subsequent generations. The results revealed that in the *Osrad51-1* mutant there is a single ‘A’ insertion in the 8th exon of *OsRAD51A1* and *OsRAD51A2* (Figure 1B, Supplementary Figure S2A,B). The *Osrad51a1* and *Osrad51a2* mutants were obtained from descendants of the original heterozygous *Osrad51-1* mutant. In the *Osrad51-2* mutant, there is a 5-nucleotide deletion in the 5th exon of *OsRAD51A1* and a 7-nucleotide deletion in the 4th exon of *OsRAD51A2* (Supplementary Figure S3A). The mutation sites in *OsDMC1A* and *OsDMC1B* are a ‘T’ insertion in the 2nd exon of both genes (Figure 1B, Supplementary Figure S2C,D). All of these mutations are predicted to result in a translational frameshift.

### 2.2. The Meiotic Process Is Disturbed in Osrad51

Both *Osrad51a1* and *Osrad51a2* exhibited normal vegetative growth and fertility (Supplementary Figure S4A–C). However, the *Osrad51* plants were completely sterile, although they exhibited normal vegetative growth and development (Supplementary Figure S4D). Pollen grains from *Osrad51a1* and *Osrad51a2* were round (Supplementary Figure S4G,H), however, pollen grains from *Osrad51* plants were empty and shrunken (Supplementary Figure S4I). Thus, OsRAD51A1 and OsRAD51A2 are functionally redundant in the rice reproductive process. In addition, the *Osdmc1* mutant and *Osrad51 Osdmc1* mutant showed normal vegetative growth but were sterile (Supplementary Figure S4E,F,K,L).

**Figure 2:**
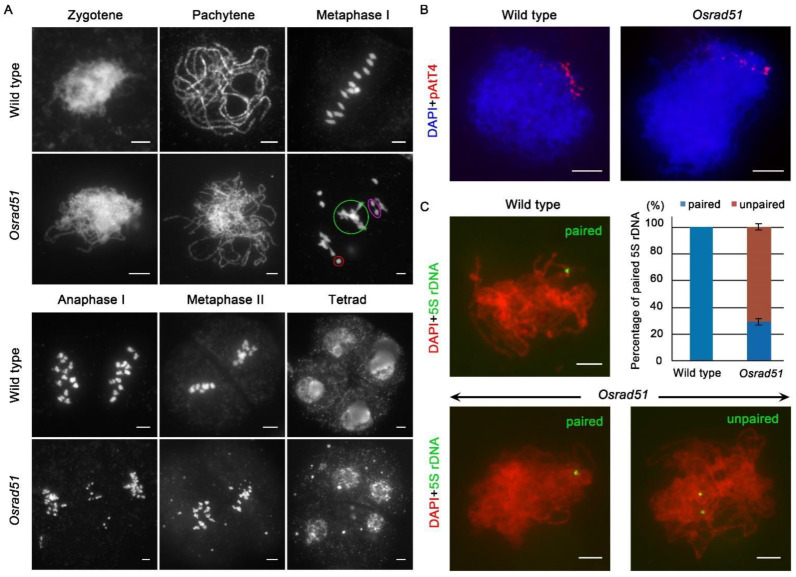
*OsRAD51* deficiency causes severe meiotic chromosomal defects. (**A**). Meiotic chromosome behavior in both wild-type and *Osrad51* PMCs. The red circle indicates univalent; the green circle indicates multivalent; the purple circle shows a separating bivalent. (**B**). Bouquet formation was analyzed using FISH with the telomere-specific pAtT4 probe (red) in the wild-type and *Osrad51* PMCs. Chromosomes were stained with DAPI (4′,6-diamidino-2-phenylindole, blue). (**C**). Chromosome pairing status in the wild-type and *Osrad51* mutant. The probe was 5S rDNA (green). Chromosomes were stained with DAPI (red). Upper left panel, fully paired pachytene chromosomes in wild type; upper right panel, histogram of observed cells according to their pairing status; lower left panel, the *Osrad51* PMCs show paired probe signals; lower right panel, the *Osrad51* PMCs show unpaired probe signals. Bars, 5 μm.

To explore the causes of the sterility in the *Osrad51* mutant, we analyzed the meiotic chromosome behaviors of pollen mother cells (PMCs) from wild-type and *Osrad51* mutant plants. In wild-type PMCs, meiotic chromosomes began to pair at zygotene (Figure 2A). Perfectly synapsed chromosomes were presented as thick threads at pachytene. At metaphase I, the further condensed bivalents aligned on the equatorial plate. Homologous chromosomes separated at anaphase I. In the second meiotic division, the equational segregation of sister chromatids occurred, and tetrads were formed (Figure 2A).

In *Osrad51* PMCs, a partially asynaptic phenotype was observed at the zygotene-pachytene transition. Differences between *Osrad51* and the wild type were evident at metaphase I. Contrary to the 12 well-defined bivalents observed in wild-type PMCs, univalents and multivalent associations were displayed in *Osrad51* PMCs (n = 35 for PMCs). At anaphase I, chromosome bridges and chromosome fragments were observed as a result of the presence of univalents and multivalents at metaphase I (n = 40 for PMCs), and abnormal tetrads with scattered DNA eventually formed in the *Osrad51* mutant (n = 30 for PMCs) (Figure 2A). Further cytological observations revealed that the meiotic defects in *Osrad51-2* were similar to those observed in *Osrad51-1* (Supplementary Figure S3B). These results suggest that the sterility of *Osrad51* plants is caused by meiotic defects.

### 2.3. The Osrad51 Mutant Shows Defects in Homologous Pairing and Synapsis

During early prophase I, telomeres attach to the inner nuclear envelope and form a cluster. Telomere bouquet clustering is essential for homologous pairing [29,30]. To detect bouquet formation during meiosis, we adopted fluorescence in situ hybridization (FISH) analysis of wild-type and *Osrad51* PMCs using the pAtT4 probe, which recognizes telomere-specific sequences [31]. In wild-type PMCs, telomeres were clustered within a confined region at early zygotene. There were no differences between *Osrad51* PMCs and the wild-type (n = 30 for PMCs) (Figure 2B). These results suggest that bouquet formation is independent of OsRAD51.

To examine chromosome pairing behavior, FISH was performed on *Osrad51* PMCs employing 5S rDNA as a specific probe. 5S rDNA is a tandemly repeated sequence located on the short arm of chromosome 11 close to the centromere in rice. During early pachytene, two side-by-side 5S rDNA signals were observed in the wild-type (n = 25 for PMCs), indicating that homologous pairing was completed between homologous chromosomes. In the *Osrad51* mutant, only 31.4% of the detected meiocytes showed paired signals for the 5S probe, and 68.6% of PMCs displayed unpaired signals (n = 70 for PMCs) (Figure 2C). These results suggest that mutation of *OsRAD51* leads to deficient homologous chromosomal pairing during rice meiosis.

**Figure 3:**
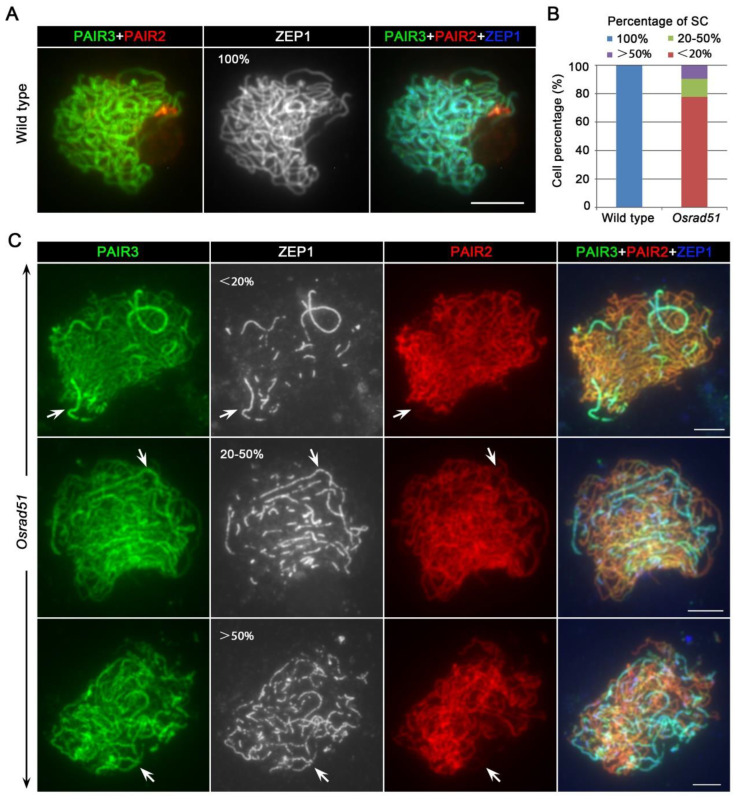
*OsRAD51* is required for the full-length homologous synapsis in rice. (**A**). Triple color immunostaining of PAIR2 (red), PAIR3 (green), and ZEP1 (blue) in wild type. (**B**). Histogram of cells according to their proportion of synapsed regions in wild type and *Osrad51*. (**C**). Triple color immunostaining of PAIR2 (red), PAIR3 (green), and ZEP1 (blue) in *Osrad51* mutant PMCs at pachytene. Arrows indicate the regions where PAIR2 signals disappeared that are exactly where ZEP1 is loaded. Bars, 5 μm.

We also detected the assembly status of the synaptonemal complex (SC) in *Osrad51* PMCs via an immunolocalization assay employing antibodies against PAIR3, PAIR2, and ZEP1 protein. PAIR3, an axial protein, is essential for homologous pairing [32]. PAIR2 is associated with axial elements proteins, and is released quickly from the chromosomal axes when SC formation is completed. ZEP1 can be used to monitor synapsis in rice [33]. In wild-type PMCs, ZEP1 signals were fully elongated along the completely paired homologous chromosomes at pachytene (Figure 3A). PAIR2 staining presented punctate signals at leptotene. The PAIR2 signals decreased gradually from synaptic chromosomes during zygotene and diminished from chromosomes after synapsis was completed at pachytene [34]. In *Osrad51* PMCs, we detected that the PAIR2 signals vanished from the regions where ZEP1 was loaded (n = 30 for PMCs) (Figure 3C, indicated by arrows). In order to detect the location pattern of PAIR2 on *Osrad51* chromosomes in more detail, we used an immunolocalization assay combined with a super-resolution structured illumination microscope (SIM). The results showed that the PAIR2 signal disappeared from the synapsed regions (n = 35 for PMCs) (Supplementary Figure S5). We then analyzed 63 *Osrad51* pachytene PMCs to measure the level of the SCs (the ratio of the length of ZEP1 to the length of PAIR3) [35,36]. The results showed that 77.8% of the PMCs presented punctuate or short liner ZEP1 signals. Of these, the SC level was <20%. In addition, 12.7% of the PMCs displayed slightly longer ZEP1 stretches, and the SC level was 20–50%. Some long ZEP1 tracks were present along the chromosomes in 9.5% of the PMCs, and the SC level was >50% (Figure 3B). Taken all together, these results suggest that the function of OsRAD51 is pivotal for the full-length synapsis.

### 2.4. Crossover Formation Is Largely Inhibite in the Osrad51 Mutant

One of the main cytological defects observed in the *Osrad51* mutant is the decreased frequency of bivalents (Figure 2A). We inspected the generation of bivalents in *Osrad51*. Unlike the 12 bivalents in the wild type, the *Osrad51* PMCs produced one to two bivalents at metaphase I. The mean number of bivalents was decreased in *Osrad51* (1.9 ± 0.2, n = 35 for PMCs). Crossovers are essential for stable bivalents formation, so the results suggest that crossover formation is probably suppressed in the *Osrad51* mutant. 

**Figure 4:**
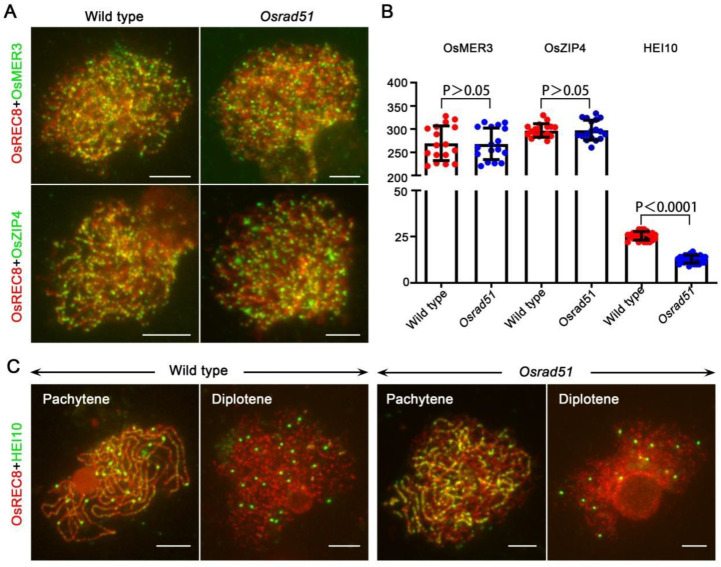
Crossover formation is suppressed in the *Osrad51* mutant. (**A**). Immunolocalization of OsMER3 (green) and ZIP4 (green) at early prophase I stage in wild type and the *Osrad51* mutant. (**B**). Quantification of OsMER3, OsZIP4, and HEI10 foci per meiocyte in wild type and *Osrad51*. Horizontal lines in the panels indicate the mean values. Statistical significance was calculated using the two-tailed Student’s *t*-test. (**C**). Immunolocalization of HEI10 (green) at late pachytene and diplotene in wild type and *Osrad51*. Bars, 5 μm.

We performed immunolocalization assays in *Osrad51* employing antibodies against OsMER3, OsZIP4, and OsHEI10, three proteins that all participate in crossover formation [37,38,39]. OsREC8 was used to indicate meiotic chromosomes, which is a conserved component of the cohesion complex [40]. The number of OsMER3 foci in the wild type (269.4 ± 9.1; n = 17 for PMCs) was comparable to that of the *Osrad51* mutant (267.9 ± 8.3, n = 17 for PMCs) at zygotene. The number of OsZIP4 foci in the *Osrad51* mutant (297.6 ± 5.1, n = 18 for PMCs) was also almost equal to that in the wild type (296.9 ± 3.4, n = 18 for PMCs) (Figure 4A,B). A previous study showed that HEI10 can indicate the Class I COs [39]. In the wild type, bright HEI10 foci were detected (25.4 ± 0.47, n = 25 for PMCs) at late pachytene, and the foci could also be detected at diplotene (Figure 4B,C). Compared to wild-type, the emergence of the bright dot-like HEI10 foci was delayed in the *Osrad51* mutant. At late pachytene, most OsHEI10 signals appeared as sparser foci, and this may be the result of delayed SC assembly. At diplotene, the prominent HEI10 foci were significantly decreased in *Osrad51* (12.8 ± 0.43; n = 25 for PMCs) (Figure 4B,C). Taken together, these results suggest that OsRAD51 can affect the crossover formation. 

### 2.5. OsRAD51 Is Dynamically Localized on Chromosomes during Early Prophase I

**Figure 5:**
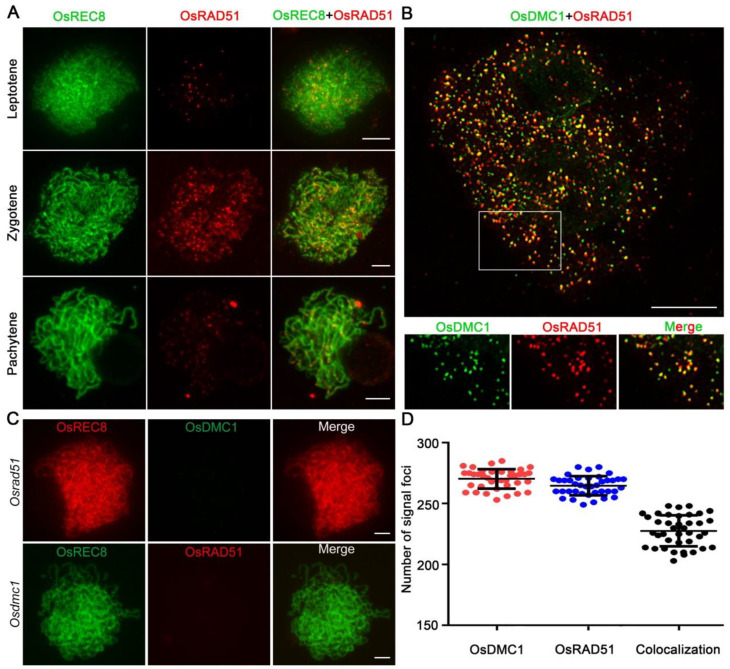
OsRAD51 can co-localize with OsDMC1. (**A**). Immunolocalization of REC8 (green) and OsRAD51 (red) in wild-type PMCs from leptotene to pachytene. Bars, 5μm. (**B**). Immunostaining of OsDMC1 (green, from mouse) and RAD51 (red, from rabbit) in wild-type PMCs at pachytene. The OsRAD51 signals were co-localized with the OsDMC1 signals, exhibiting bright yellow signal points. Magnified images of the boxed region are shown in the panels below. Bars, 5 μm. (**C**). The OsRAD51 signal cannot be detected in *Osdmc1*, and the OsDMC1 signal cannot be detected in *Osrad51*. Bar, 5 μm. (**D**). Quantification of the co-localized signal foci of OsDMC1 and OsRAD51 per PMC in the wild type (n = 39).

To investigate the specificity of the OsRAD51 antibody, we conducted an immunolocalization assay in wild-type PMCs and *Osrad51* mutant PMCs. No OsRAD51 signals were detected in *Osrad51* mutant PMCs but were detected in the *Osrad51a1* and *Osrad51a2* mutants (Supplementary Figure S6A). In addition, results of the western-blot assay reveal that the OsRAD51 antibody could clearly recognize OsRAD51 in total extracts from the young panicles of the wild type, *Osrad51a1*, and *Osrad51a2*, but no signal was detected in the protein sample of *Osrad51* (Supplementary Figure S6B). These results suggest that the antibody directed against RAD51 can recognize OsRAD51A1 and OsRAD51A2 simultaneously. To inspect the spatial and temporal localization of OsRAD51 during rice meiosis, we performed a dual immunolocalization assay in wild-type PMCs using specific antibodies directed individually against OsREC8 and OsRAD51. In the wild-type PMCs, OsRAD51 signals firstly emerged as punctate foci on chromosomes at leptotene, gradually appearing as high-intensity dot-like signals on zygotene chromosomes. During pachytene, the signals gradually became weak (Figure 5A). 

Meiotic recombination is induced by the programmed formation of DSBs that is catalyzed by Spo11, a functional homolog of topoisomerase-like protein. To determine whether the loading of OsRAD51 depends on the formation of DSBs, we carried out a dual immunolocalization assay in *spo11-1* PMCs using antibodies against OsRAD51 and OsREC8. There were no obvious OsRAD51 signals in *Osspo11-1* PMCs, which suggests that the loading of OsRAD51 depends on meiotic DSB formation (Supplementary Figure S7A). γH2AX is an isoform of histone H2A.γH2AX is a reliable cytological marker for detecting DSB in most eukaryotes [41]. Therefore, we performed dual immunolocalization assays in wild-type and *Osrad51* PMCs employing polyclonal antibodies specifically against γH2AX and OsREC8 individually. In wild-type PMCs, γH2AX signals emerged as dots and patchy areas at zygotene. In the *Osrad51* mutant, the pattern of γH2AX signals was comparable to that of the wild type (Supplementary Figure S7B), suggesting that DSB formation is not affected in *Osrad51*.

### 2.6. OsRAD51 and OsDMC1 Are Co-Localized and Functionally Interdependent

To further investigate the relationship between OsRAD51 and OsDMC1, we conducted a dual immunolocalization assay in wild-type PMCs using specific antibodies against OsRAD51 and OsDMC1 simultaneously. The results of super-resolution SIM on wild-type PMCs revealed that the OsRAD51 signals co-localized with OsDMC1 as bright yellow foci (Figure 5B). Furthermore, quantitative co-localization analysis revealed that the co-located signals accounted for 84% of the OsDMC1 foci and 86% of OSRAD51 foci (n = 39 for PMCs) (Figure 5B,D). These results show that OsRAD51 had considerable colocalization with OsDMC1. Immunostaining for OsRAD51 and OsDMC1 was also performed in the *Osdmc1* and *Osrad51* mutants, respectively. There were no obvious OsRAD51 signals on *Osdmc1* meiotic chromosomes, and the OsDMC1 signal could not be detected in the *Osrad51* mutant (n = 30 for PMCs) (Figure 5C). These results indicate that the loading of OsRAD51 and OsDMC1 may be interdependent.

**Figure 6:**
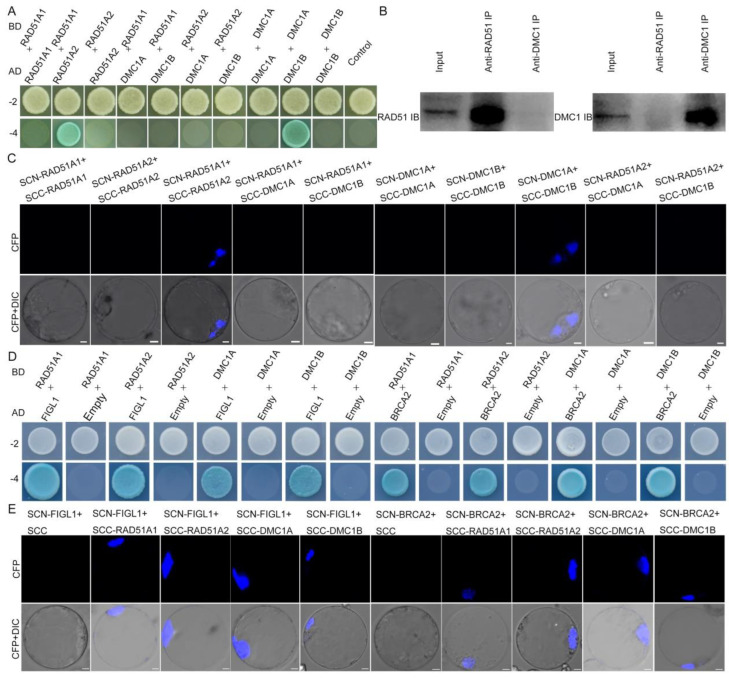
OsRAD51 cannot interact with OsDMC1, both OsRAD51 and OsDMC1 can interact with OsFIGL1 and OsBRCA2. (**A**). OSRAD51 cannot interact with OsDMC1 in yeast-two-hybrid (Y2H) assays. Interactions between bait and prey were examined on the control medium 2 (SD/−Leu/−Trp) and the selective medium 4 (SD/−Ade/−His/−L−u/−Trp). AD, prey vector pGADT7; BD, bait vector pGBKT7. (**B**). Coimmunoprecipitation assays showing that OsRAD51 cannot interact with OsDMC1. IB, immunoblot; IP, immunoprecipitation. (**C**). Bimolecular fluorescence complementation (BiFC) assays showing that OsRAD51 cannot interact with OsDMC1 in rice protoplasts. (**D**). OsRAD51 and OsDMC1 can interact with OsFIGL1 and OsBRCA2 in Y2H assays. (**E**). Bimolecular fluorescence complementation (BiFC) assays showing that OsRAD51 and OsDMC1 can interact with OsFIGL1 and OsBRCA2 in rice protoplasts. Bars, 5 μm.

To determine whether OsRAD51 interacts with OsDMC1, we carried out yeast two-hybrid (Y2H) and BiFC assays. The results revealed that OsRAD51 cannot interact with OsDMC1 (Figure 6A,C). In order to verify this result, we implemented CoIP assays. Total proteins were extracted from rice panicles in the meiosis stage. The immunoprecipitates of anti-OsRAD51 were detected by anti-OsDMC1 and the immunoprecipitates of anti-OsDMC1 were detected by anti-OsRAD51. These results showed that OsRAD51 does not interact with OsDMC1 directly (Figure 6B). We also found that OsRAD51A1 can interact with OsRAD51A2, and OsDMC1A can interact with OsDMC1B, but OsRAD51A1, OsRAD51A2, OsDMC1A, and OsDMC1B cannot interact with themselves (Figure 6A,C). In addition, we found that both of these recombinases can interact with OsFIGL1 and OsBRCA2 (Figure 6D). BRCA2 was previously reported to be a positive accelerator in controlling the dynamics of RAD51 and DMC1 focus formation, whereas FIGL1 is a negative regulator in Arabidopsis [42]. We further confirmed these interactions using BiFC assays (Figure 6E). These results imply that BRCA2 and FIGL1 may mediate the functions of these two recombinases in rice meiotic homologous recombination.

### 2.7. Chromosomal Behavior in the Osrad51 Osdmc1 Mutant Is Similar to That in the Osrad51 Mutant

**Figure 7:**
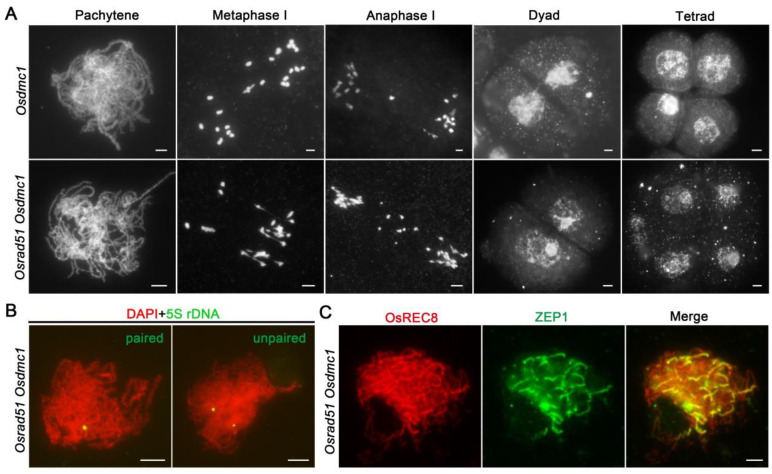
Genetic analysis of *Osrad51* with *Osdmc1*. (**A**). Meiotic chromosome behavior in the *Osrad51 Osdmc1* double mutant PMCs. (**B**). The chromosome pairing status in *Osrad51 Osdmc1*. The painting probe was 5S rDNA. Chromosomes were stained with DAPI (red). Left panel, the *Osrad51 Osdmc1* PMCs show paired probe signals; right panel, the *Osrad51* PMCs show unpaired probe signals. (**C**). Immunostaining for a transverse filament protein of the SC (ZEP1, green) in the *Osrad51 Osdmc1* double mutant at pachytene. OsREC8 signals (red) show the chromosomes. Bars, 5 μm.

Meiosis is disrupted in both the *Osrad51* and *Osdmc1* mutants, but the former displays univalents and multivalent chromosome associations, whereas the latter shows ~24 univalents at metaphase I (Figure 7A), and this result is consistent with previous articles [28]. To further investigate in depth the relationship between the two recombinases, we carried out a genetic analysis between them. The meiotic chromosomal phenotype of the *Osrad51 Osdmc1* mutant is shown in Figure 7A. In *Osrad51 Osdmc1* PMCs, univalents and multivalents were observed at metaphase I (n = 20 for PMCs). Chromosome bridges and chromosome fragmentation were detected at anaphase I (n = 20 for PMCs). At the end of meiosis, abnormal tetrads with scattered DNA were observed. Thus, chromosomal behavior in the *Osrad51 Osdmc1* mutant is similar to that of *Osrad51*.

In order to examine homologous pairing in the *Osrad51 Osdmc1*. FISH assays were performed in *Osrad51 Osdmc1* PMCs using 5S rDNA as a specific probe. During early pachytene, 7.4% of the examined PMCs showed paired or partially paired 5S rDNA signals, and 92.6% of PMCs displayed unpaired signals (n = 54 for PMCs) (Figure 7B). We further investigated SC assembly in *Osrad51 Osdmc1* PMCs via immunolocalization assays employing antibodies specifically against ZEP1. ZEP1 signals emerged as only a few short lines on chromosomes at pachytene (Figure 7C). A previous study reported that although homolog pairing occurs in the *Osdmc1a Osdmc1b* double mutant, synapsis is deficient [28]. In addition, we also found that the location of ZEP1 appears to be abnormal in the *Osdmc1* mutant (n = 25 for PMCs) (Supplementary Figure S8). These results indicate that although OsRAD51 and OsDMC1 are essential for SC assembly, the individual functions of OsRAD51 and OsDMC1 are not identical in homologous recombination.

## 3. Discussion

### 3.1. Functional Conservation and Divergence of RAD51-Like Genes

RAD51 paralogs are conserved in eukaryotes. The yeast has four RAD51 paralogs, namely Rad51, Dmc1, Rad55, and Rad57, and humans have seven RAD51 paralogs: RAD51, DMC1, RAD51B, RAD51C, RAD51D, XRCC2, and XRCC3 [43]. In yeast, Rad55 and Rad57 form a stable heterodimer that associates with Rad51 nucleofilaments. The *rad55* and *rad57* mutants show DSB repair deficiency and DNA damage sensitivity and can be alleviated by *rad51* gain-of-function alleles [44]. In humans, the BCDX2 and the CX3 formed a complex via the RAD51 paralogs [45].

In addition to *RAD51* and *DMC1*, there are five *RAD51* paralogs, including *RAD51B*, *RAD51C*, *RAD51D*, *XRCC2*, and *XRCC3* in Arabidopsis and rice. In Arabidopsis, mutations in *AtRAD51*, *AtRAD51C*, or *AtXRCC3* cause the presence of entangled chromosomes [18,46,47,48], while AtRAD51B, AtRAD51D, and AtXRCC2 seem to be unessential for meiotic DSB repair, because chromosomal behavior is normal in the triple mutant, and the triple mutant is fertility [49,50]. These proteins have partially functional redundancy in mitotic DNA repair [50]. Interestingly, in rice, the *Osrad51d* mutant displays abnormal homologous chromosome pairings and fragmented chromosomes [51,52], which indicates that the functions of *RAD51* paralogs in meiotic HR have differentiated during evolution between rice and Arabidopsis.

### 3.2. The Role of RAD51 in Meiosis

In yeast, the *rad51* mutant is hypersensitive to ionizing radiation and DNA-damaging chemicals [14]. Deletion of RAD51 in the nematode (*Caenorhabditis elegans*) leads to failure of chiasma formation and 12 properly condensed univalents at diakinesis, but pairing and synapsis are normal [53]. In the *Drosophila melanogaster rad51* mutant, synapse formation was found to be appropriately initiated, but the resolution of synapsis was delayed [54]. Mutation of RAD51 in *Tetrahymena thermophila* causes an arrest early in meiosis I [55]. However, in mice and chicken, the *rad51* null mutation is lethal [15,56]. Furthermore, the number of RAD51 foci is decreased in many maize mutants that are defective in pairing [23]. In Arabidopsis, RAD51 is not essential for vegetative and floral development, but the *Atrad51* mutant displays severe meiotic defects, including partial homologous pairing and synapse, chromosome fragmentation, and multivalent chromosome associations [18,20]. Our data show that RAD51 is essential for normal meiosis in rice, but is not required for vegetative development. In contrast to the wild type, *Osrad51* chromosomes are not paired perfectly at zygotene, SC formation is deficient. Moreover, univalents and multivalent associations are present at metaphase I, and chromosome fragments and unbalanced chromosome segregation are detected at anaphase I. Chromosome fragmentation may be the reason behind the abnormal segregation of chromosomes. The problems in homologous pairing and synapsis suggest that RAD51 is important for interhomolog recognition during meiosis. Marta et al. [57] claimed that DSBs were repaired in the RAD51-dependent manner, presumably using the sister chromatid as a template in meiotic recombination. Lambert et al. [58], reported that Rad51 is mostly responsible for homologous recombination between sister chromatids in somatic cells. Xu et al. [25] also revealed that RAD51 paralogs play essential roles in somatic homologous recombination for DNA repair. Taken together, RAD51 has different influences on meiosis in different organisms, and the function of RAD51 may be slightly different between mitosis and meiosis.

### 3.3. Functional Relationship between OsRAD51 and OsDMC1 in Meiosis

Once generated by Spo11, DSBs are processed by 5′ nucleolytic resection to yield 3′ single strands DNA (ssDNA) tails. Then, these DNA overhangs are coated by RAD51 and DMC1 [13]. The recombinases bind to the 3′ ssDNA forming presynaptic filaments, which then invade double-stranded DNA to generate a heteroduplex [59]. The presence of meiosis-specific proteins that bond DNA strand exchange to homologous chromosomes makes meiotic HR different from mitotic HR. Previous studies have suggested that DMC1 and its cofactors specifically participated in the interhomolog bias [60]. Nevertheless, the exact way both recombinases work remains unclear. RAD51 plays a pivotal non-enzymatic role, facilitating DMC1 assembly and guiding it to invade a homologous chromosome rather than a sister chromosome [61,62]. Therefore, DMC1 is the dominant meiotic strand-exchange enzyme [62,63].

In Arabidopsis, Kurzbauer reported that the number of DMC1 foci was sharply decreased in the *Atrad51* mutant, whereas the number of RAD51 foci in *Atdmc1* has no change compared to the wild type. They also confirmed that AtRAD51 and AtDMC1 cannot co-localize during any meiotic stage, and they declared that AtRAD51 and AtDMC1 occupy opposite DNA ends at a DSB [64]. Chen recently showed that AtRAD51 promotes AtDMC1 localization on meiotic chromosomes by inhibiting the SMC5/6 complex, while the SMC5/6 complex inhibits AtDMC1 during meiotic recombination. These authors confirmed that AtRAD51 interacts with AtDMC1 [24]. In rice, it is interesting that OsRAD51 cannot load onto *Osdmc1* mutant chromosomes, and OsDMC1 cannot load onto *Osrad51* mutant chromosomes. We also found that most OsRAD51 foci can co-localize with OsDMC1. Thus, we can draw the conclusion that the loading of OsRAD51 and OsDMC1 may be interdependent, and they may be functionally interdependent in rice meiotic recombination. Meiosis is abnormal in both the *Osrad51* and *Osdmc1* mutants, *Osrad51* displays univalents, bivalents, and multivalents at metaphase I, and some chromosome fragmentation was observed at anaphase I, which could be the result of wrongly repaired DSBs, whereas *Osdmc1* shows ~24 univalents, the sister chromatid was used as the template to repair meiotic DSBs, and avoid the production of the chromosome fragmentation. Those results suggest that these two recombinases may play different roles in DNA repair. The meiotic chromosome behavior in the *Osrad51 Osdmc1* mutant indicates that OsDMC1 may function in close collaboration with OsRAD51 in rice meiotic recombination. Yeast two-hybrid analysis, CoIP, and BiFC assays demonstrated that OsRAD51 cannot interact directly with OsDMC1. However, we found that both OsRAD51 and OsDMC1 can interact with OsFIGL1 and OsBRCA2. Kumar reported that BRCA2 is a positive accelerator that provides the stabilizing functions and FIGL1 acts as a negative regulator to disassemble the filaments in Arabidopsis [41]. Fu also confirmed that OsBRCA2 is indispensable for facilitating the loading of OsRAD51 and OsDMC1 onto 3′ ssDNA of programmed DSBs during meiosis to facilitate single-end invasions of homologous chromosomes and precise recombination in rice [65]. Our data suggest that other proteins may mediate the functions of these two recombinases during rice meiosis. Although DMC1 and RAD51 are highly functional conserved in eukaryotes, there may be some differences between species. These results offer further insight into the inter-relationship between OsRAD51 and OsDMC1.

## 4. Materials and Methods

### 4.1. Vector Construction and Plant Cultivation

A multi-gene editing system for rice *RAD51* and *DMC1* was constructed. In this system, expression of the Cas9 gene was driven by the CaMV 35S promoter, and four sgRNA expression cassettes, which were driven by OsU3 promoters, were arranged in tandem. For target recognition (Supplementary Table S1 for the target sequences), 20-nt guide oligo-nucleotides were synthesized with appropriate adaptor sequences for seamless ligation with the promoter fragments. The tandem sgRNA expression cassettes were first constructed in the pUC19 intermediate vector, and the resultant DNA fragment was constructed into the pCAMBIA1300 backbone with the Cas9 expression cassette. The constructs were transformed into the calli of the *japonica* rice variety “Yandao 8” using *Agrobacterium*-mediated transformation. Other mutant materials were obtained from our previous study. All plant materials were grown in paddy fields. The genotypes were identified by PCR in the F_2_ population derived from self-pollination of heterozygous F_1_ plants. The sequences of the oligonucleotide primers used in this study are given in Supplementary Table S1.

### 4.2. Real-Time PCR for Gene Expression Analysis

Total RNA was separately extracted from the leaf, root, internode, and panicle of wild-type plants. The Bio-Rad CFX96 real-time PCR instrument was used to perform the real-time PCR assays. EvaGreen (Biotium) was used as the fluorescent dye for RT-PCR. The real-time PCR was performed with the specific primers RT-RAD51A1F, RT-RAD51A1R, RT-RAD51A2F, and RT-RAD51A2R for *OsRAD51* as well as *Actin*F and *Actin*R for the rice *ACTIN* gene. The primer sequences are given in Supplementary Table S1. Each experiment was replicated three times. The experimental results were analyzed using Bio-Rad CFX Manager analysis software.

### 4.3. Immunofluorescence Analysis

Fresh young panicles were first fixed in 4% (*w*/*v*) paraformaldehyde for 30 min at room temperature. Chromosome preparation and immunofluorescence analysis were conducted as previously described [37]. The immunofluorescence analysis used the following primary antibodies: rabbit antibodies directed against OsREC8 [39], PAIR2 [31], and OsRAD51 [25], and mouse antibodies directed against OsREC8 [40], PAIR3, ZEP1 [33], OsZIP4 [38], OsMER3 [37], OsDMC1 [28], and OsHEI10 [39] individually. All of the above antibodies were previously generated in our laboratory. Additionally, a 561-bp fragment of the *PAIR3* cDNA encoding amino acids 248 to 393 was cloned into the vector pGEX4T-2. The anti-PAIR3 guinea pig antibody was generated with the PAIR3 fusion protein. The secondary antibodies, including rhodamine-conjugated goat anti-rabbit antibody, fluorescein isothiocyanate-conjugated sheep anti-mouse antibody, and AMCA-conjugated goat anti-guinea pig antibody, were used for fluorescent detection. Images were captured with a Zeiss A2 fluorescence microscope equipped with a micro CCD camera. The super-resolution images were captured using a DeltaVision microscope (OMX^TM^ V4; GE Healthcare, Chicago, IL, USA) and processed with S_OFT_W_O_R_X_ (Applied Precision, Issaquah, WA, USA) to generate projected images. Co-localization analysis was performed using an automated graphic plugin running IMAGE J 1.37a software (http://wwwfacilities.uhnresearch.ca/wcif, accessed on 2 March 2021) as described by Li et al. [66].

### 4.4. Cytological Procedures and Data Analysis

Young panicles containing pollen mother cells (PMCs) entering meiosis were fixed in Carnoy’s solution (ethanol:glacial acetic acid, 3:1) and stored at −20 °C to be used in chromosome spreads. PMCs at the meiotic stage were squashed on slides and stained with acetocarmine. The squashes were then covered with coverslips on the slides, which were then rapidly frozen in liquid nitrogen. The samples were dehydrated through an ethanol series (70, 90, and 100%) after the coverslips were removed. DAPI (4′,6-diamidino-2-phenylindole) dissolved in an antifade solution (Vector Laboratories) was used to counterstain the chromosomes on the slides [37].

### 4.5. Yeast Two-Hybrid Assay

To perform Yeast two-hybrid assay, *OsRAD51A1*, *OsRAD51A2*, *OsDMC1A*, *OsDMC1B*, *OsFIGL1*, and *OsBRCA2* were amplified using gene-specific primers (Supplementary Table S1) with KOD-plus polymerase and then ligated into the vectors pGBKT7 and pGADT7. Plasmid vectors were transformed into the Y2H Gold yeast strain using the LiAc/PEG method, and pGBKT7 and pGADT7 were used as the bait and prey vectors, respectively.

Yeast transformants were mated on YPDA medium for 48 h and selected on SD/–Trp–Leu plates for 36 h. Transformants were then selected on SD/–His–Ade–Trp–Leu medium containing X-α-Gal and AbA to test for positive interactions.

### 4.6. BiFC Assay

To conduct BiFC assays, *OsRAD51A1*, *OsRAD51A2*, *OsDMC1A*, *OsDMC1B*, *OsFIGL1*, and *OsBRCA2* were amplified using gene-specific primers (Supplementary Table S1) with KOD-plus polymerase and then ligated into the BiFC vectors pSCYNE (SCN) and pSCYCE(R) (SCC) [67]. The plasmid constructs were transformed into protoplasts as described by Bart et al. [68]. After incubation at 28 °C in the dark for 18 h, the CFP signals were captured with a confocal laser scanning microscope at an excitation wavelength 405 nm (Leica TCS SP5).

### 4.7. CoIP Assay

Total proteins were extracted from rice panicles in meiosis stage with extraction buffer (50 mM Tris-HCl, pH 7.5, 150 mM NaCl, 0.5% NP40, 1 mM phenylmethylsulfonyl fluoride, 1 × protease inhibitor cocktail). The lysates were incubated with Anti-OsRAD51 or Anti-OsDMC1. The Dynabeads™ Co-Immunoprecipitation Kit (Invitrogen, 14321D, Waltham, MA, USA) was used and Co-IP analysis was performed based on the technical bulletin. The immunoprecipitates were electrophoretically separated by SDS-PAGE and transferred to a polyvinylidene fluoride (PVDF) membrane (GE Healthcare). Proteins were detected by treating the membranes with anti-OsRAD51 (1:1000) or anti-OsDMC1 (1:1000).

### 4.8. Western-Blot Assay

Total proteins were separately extracted from wild type, *Osra51a1* and *Osrad51a2* rice panicles in meiosis stage with extraction buffer (50 mM Tris-HCl, pH 7.5, 150 mM NaCl, 0.5% NP40, 1 mM phenyl methylsulfonyl fluoride, 1 × protease inhibitor cocktail). Protein samples were separated by SDS-PAGE on a 12% polyacrylamide gel and electroblotted onto polyvinylidene difluoride membrane (Amersham). Western blots were carried out to detect the OsRAD51 protein using OsRAD51 antibodies.

### 4.9. Fluorescence In Situ Hybridization

FISH analysis was conducted according to Zhang et al. [31]. The pAtT4 (telomeric repeats) and pTa794 (5S rRNA genes from wheat) [69] clones were used as probes. Chromosomes were counterstained with DAPI. The slides were observed under a Zeiss A2 fluorescence microscope and images were captured with a DVC1412 CCD camera using IPLab4.0 software.

### 4.10. Computational and Database Analysis

Protein sequence similarity searches were performed using BLASTp (www.ncbi.nlm.nih.gov/BLAST, accessed on 2 March 2021). Gene structure schematic diagrams were generated by GSDS (http://gsds.cbi.pku.edu.cn/index.php, accessed on 2 March 2021). The multiple sequence alignment diagram was generated using GenDoc software. The lengths of ZEP1 and REC8 on chromosomes were measured with Image J software.

### 4.11. Accession Numbers

The sequences used in the alignments are available in the GenBank/EMBL databases under the following accession numbers: OsRAD51A1, BAF28642.1; OsRAD51A2, BAB85492.1; ZmRAD51A1 (*Zea mays*), NP_001104918; ZmRAD51A2 (*Zea mays*), NP_001104919, SlRAD51 (*Solanum lycopersicum*), AAC23700.1; AtRAD51 (*Arabidopsis thaliana*), AAC49555.1; PtRAD51 (*Populus trichocarpa*), XP_024459211.1; ScRAD51 (*Saccharomyces cerevisiae*), EGA79221.1; SpRAD51 (*Schizosaccharomyces pombe*), CAA80878.1; MmRAD51 (*Mus musculus*), BAA02961.1; and HsRAD51 (Homo sapiens), NP_002866.2.

## Supplementary Materials

The following supporting information can be downloaded at: https://www.mdpi.com/article/10.3390/ijms23179906/s1.
