# Supplementary material for: OsRAD51 Plays a Vital Role in Promoting Homologous Recombination in Rice Meiosis
Source: Int J Mol Sci. 2022 Aug 31;23(17):9906. doi: 10.3390/ijms23179906 (PMC9456343; doi:10.3390/ijms23179906)
Supplement: Supplementary file 1 [file ijms-23-09906-s001.zip › ijms-1864540-supplementary.pdf]

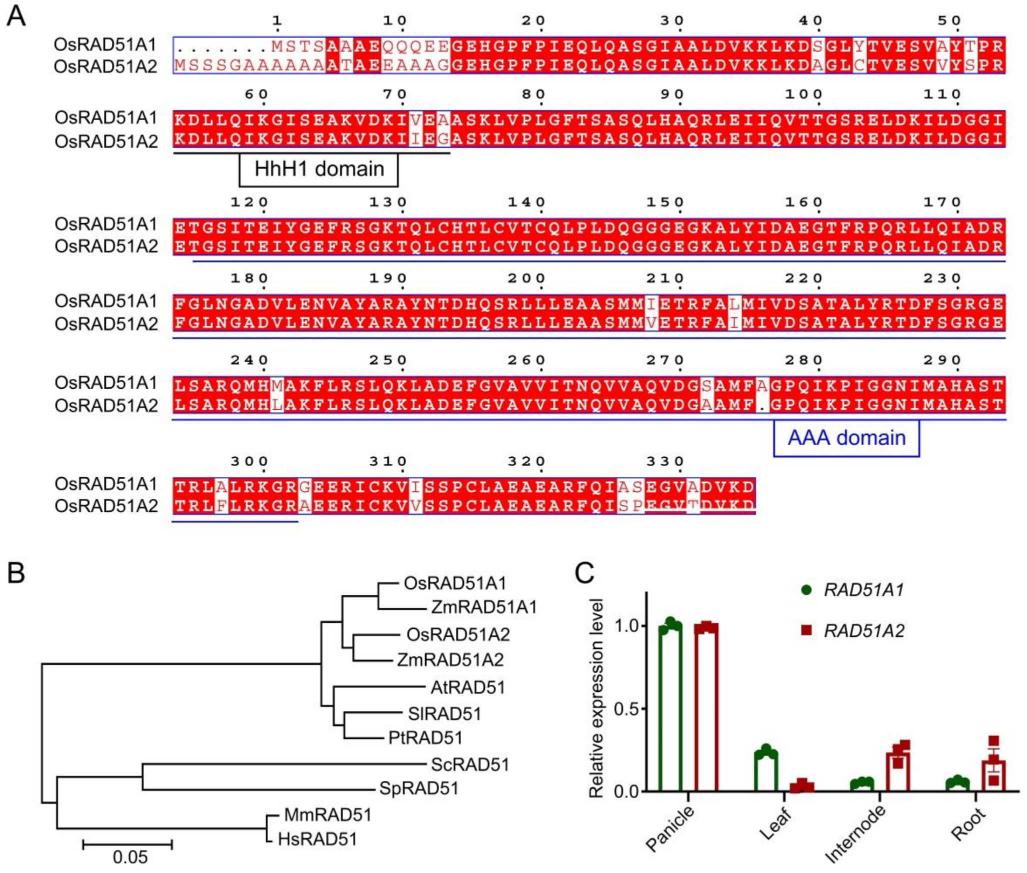

Supplementary Figure S1. Characterization of OsRAD51.

A. OsRAD51 contains an N-terminal HhH1 domain (black line) and a AAA domain (blue line). **B.** The phylogenetic tree for rice (*Oryza sativa*) OsRAD51. These proteins include OsRAD51A1 and OsRAD51A2 from *Oryza sativa*, ZmRAD51A1 and ZmRAD51A2 from *Zea mays* L., AtRAD51 from *Arabidopsis thaliana*, SIRAD51 from *Solanum lycopersicum*, PtRAD51 from *Populus trichocarpa*, ScRAD51 from *Saccharomyces cerevisiae*, SpRAD51 from *Schizosaccharomyces pombe*, MmRAD51 from *Mus musculus* and HsRAD51 from *Homo sapiens*. **C.** Real-time PCR analysis of rice (*Oryza sativa*) *OsRAD51* gene in different tissues.

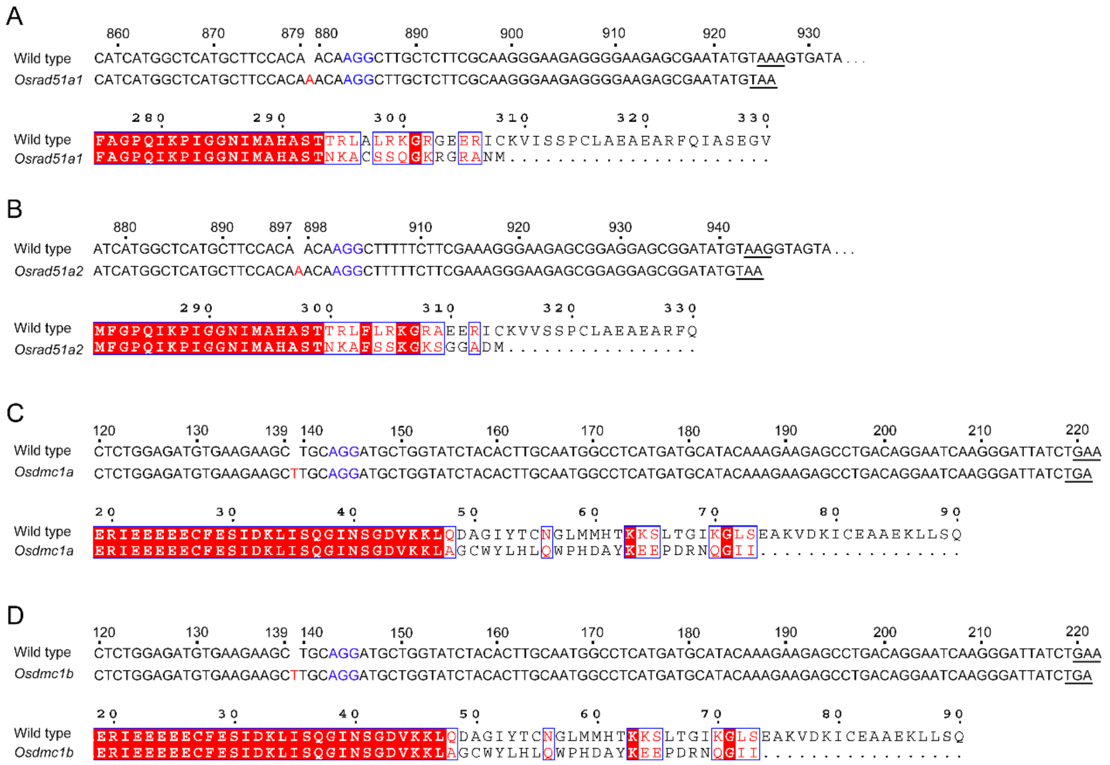

Supplementary Figure S2. The deduced proteins in the mutants.

A. The deduced proteins in *Osrad51a1* mutants. **B.** The deduced proteins in *Osrad51a2* mutants. **C.** The deduced proteins in *Osdmc1a* mutants. **D.** The deduced proteins in *Osdmc1b* mutants.

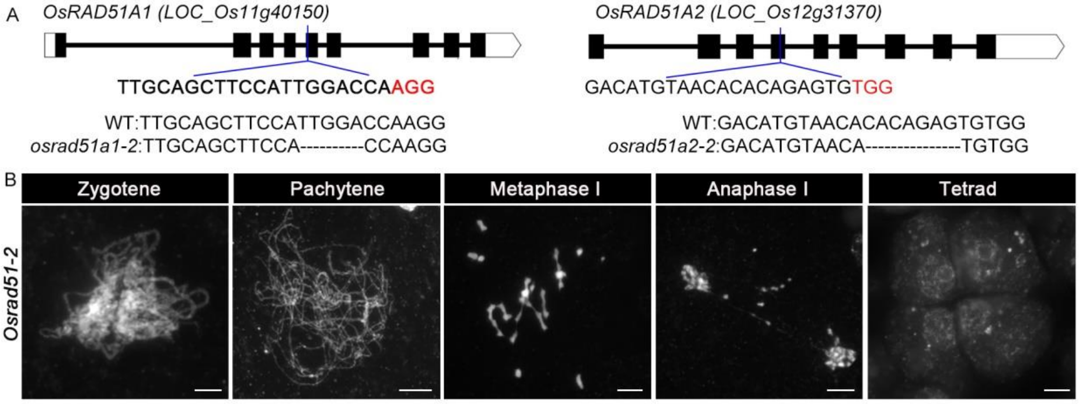

Supplementary Figure S3. Strategy of vector construction for *Osrad51-2* (A) and meiotic chromosome behaviors in *Osrad51-2* PMCs (B). Bars, 5 µm.

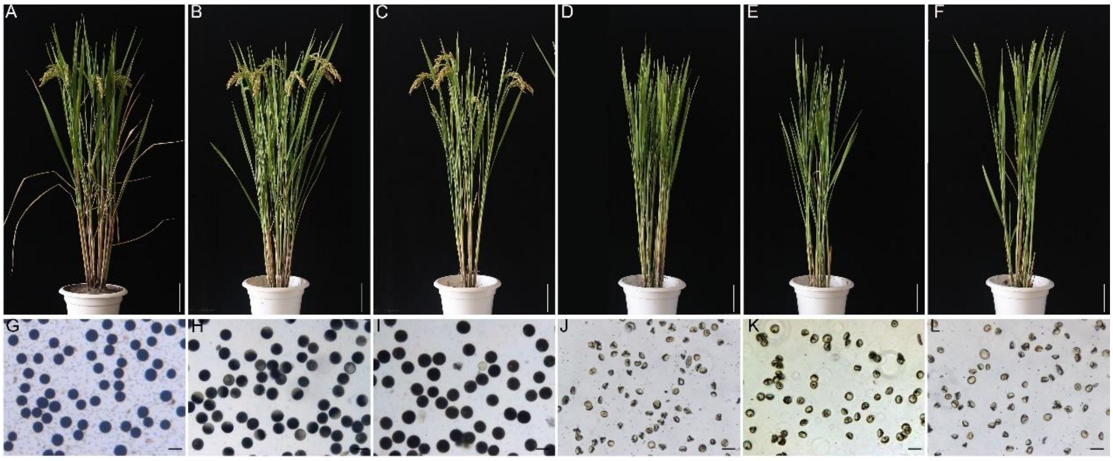

Supplementary Figure S4. The phenotype of *Osrad51*, *Osdmc1* and *Osrad51 Osdmc1*.

The representative images from WT (A), *Osrad51a1* (B), *Osrad51a2* (C), *Osrad51* (D), *Osdmc1* (E), and *Osrad51 Osdmc1* (F) mutant plant. Pollen grains stained with 1% I_2-KI solution in WT (G), *Osrad51a1* (H), *Osrad51a2* (I), *Osrad51* (J), *Osdmc1* (K), and *Osrad51 Osdmc1* (L). Bars, 10 cm (A-E), 50 μm (F-J).

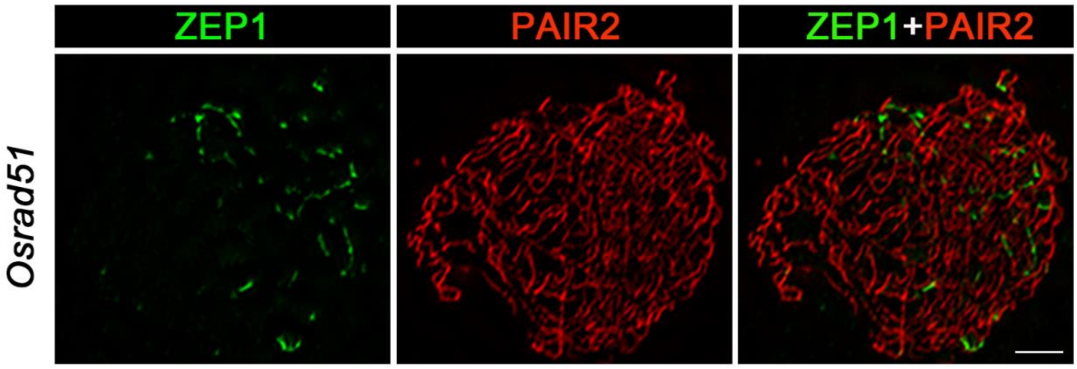

Supplementary Figure S5. PAIR2 signal disappeared from the synapsed regions in osrad51. Structured illumination microscopy is used to observe the distribution of ZEP1 (green) and PAIR2 (red) at pachytene. Bar, 5 μm.

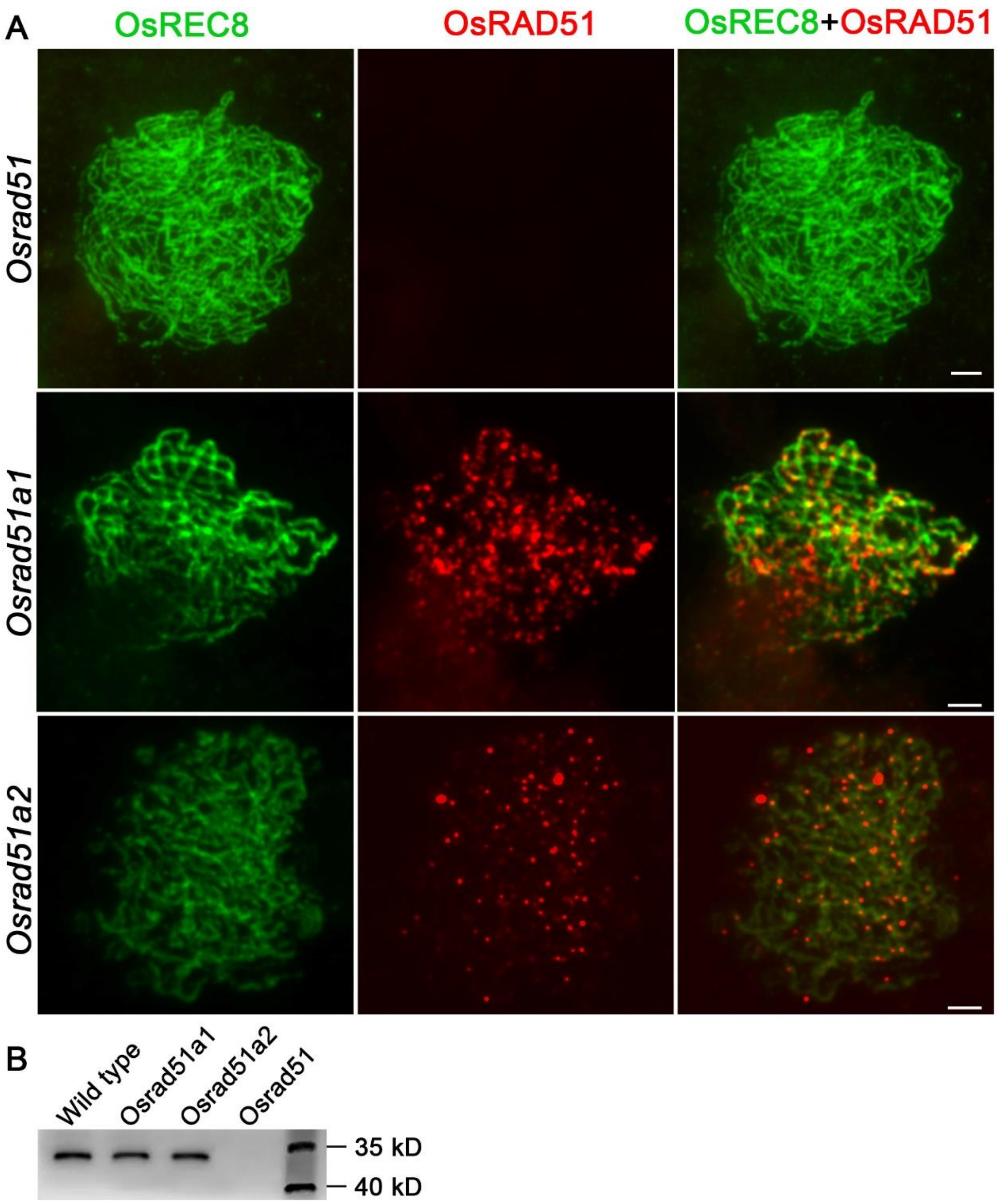

Supplementary Figure S6. The antibody against RAD51 can specifically recognize OsRAD51A1 and OsRAD51A2 simultaneously. (A) Immunolocalization of OsRAD51 in wild type and the *Osrad51* mutant. Bar, 5μm. (B) Western blot analysis using anti-OsRAD511 antibody.

.

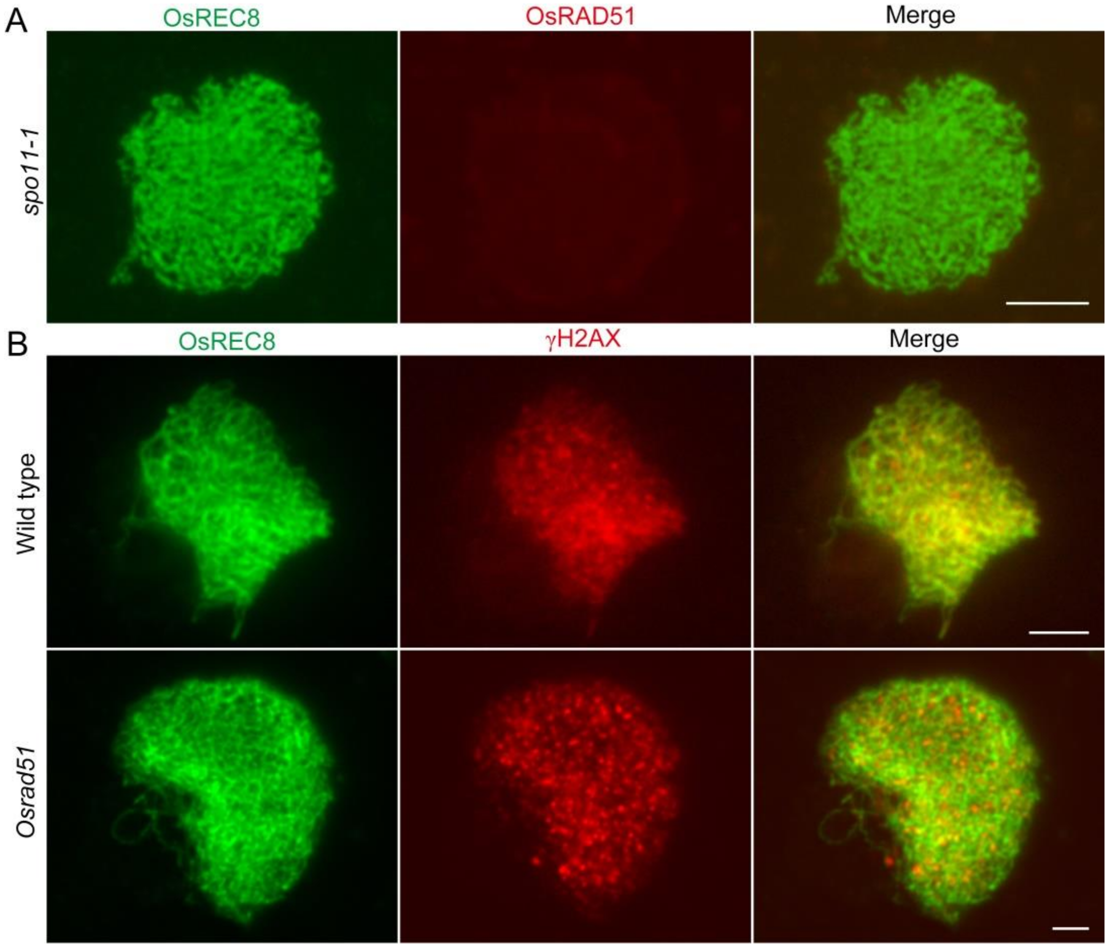

Supplementary Figure S7. The loading of OsRAD51 depend on meiotic DSB formation

A. The loading pattern of OsRAD51 in *spo11-1*. **B.** The loading pattern of γH2AX in wild type and *Osrad51*. Bars, 5μm.

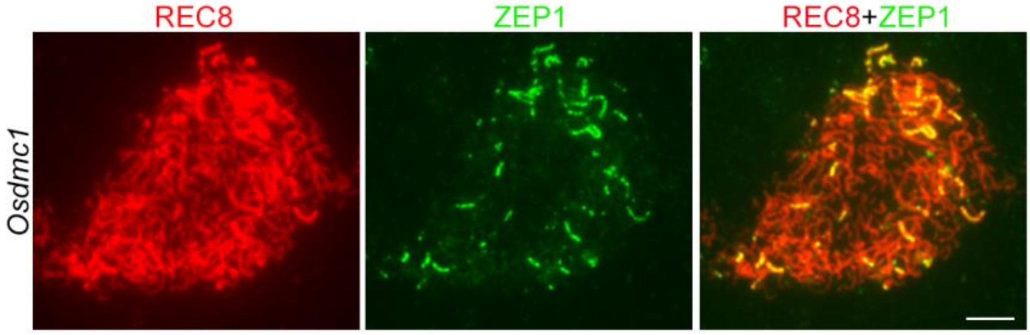

Supplementary Figure S8. The localization of ZEP1 in *Osdmc1* appeared abnormal. Bar, 5μm.

Supplementary Table S1 List of primers used in this study.

Primer names	Sequences(5'→3')	Application
RT-RAD51A1F	TCGGGTTGAATGGTGCTGATGT	PCR for *RAD51A1* quantification
RT-RAD51A1R	GTGCATTTGCCTCGCTGAGAG	PCR for *RAD51A1* quantification
RT-RAD51A2F	TGGCGTGGCGGTGGTAATCA	PCR for *RAD51A2* quantification
RT-RAD51A2R	ATATCCGCTCCTCCGCTCTTCC	PCR for *RAD51A2* quantification
ActinF	CTGACAGGATGAGCAAGGAG	PCR for *Actin* quantification
ActinR	GGCAATCCACATCTGCTGGA	PCR for *Actin* quantification
CrRAD51	TGGCTCATGCTTCCACAACAAGG	Generate Crispr/cas9 construct for *RAD51* in *Osrad51-1*
CrRAD51A1-2	TTGCAGCTTCCATTGGACCAAGG	Generate Crispr/cas9 construct for *RAD51A1* in *Osrad51-2*
CrRAD51A2-2	GACATGTAACACACAGAGTGTGG	Generate Crispr/cas9 construct for *RAD51A2* in *Osrad51-2*
CrDMC1	TGGAGATGTGAAGAAGCTGCAGG	Generate Crispr/cas9 construct for DMC1
Cr-RAD51A1-seq-F	TTGGAGTGGCTGTGGTTATCA	PCR for identifying RAD51A1 mutation in *Osrad51-1*
Cr-RAD51A1-seq-R	CAATCAGCAGGAGAGCAACAC	PCR for identifying RAD51A1 mutation in *Osrad51-1*
Cr-RAD51A1-2-seq-F	ACAACTGGATCTAGAGAGCTTGAT	PCR for identifying RAD51A1 mutation in *Osrad51-2*
Cr-RAD51A1-2-seq-R	CCAGCAGAAGTCTTGATTGATGA	PCR for identifying RAD51A1 mutation in *Osrad51-2*
Cr-RAD51A2-2-seq-F	TCTTGGATGGTAGCCTCTTGG	PCR for identifying RAD51A2 mutation in *Osrad51-2*
Cr-RAD51A2-2-seq-R	GCAGGTCAATCTTTAGCACAAC	PCR for identifying RAD51A2 mutation in *Osrad51-2*

Cr-RAD51A2-seq-F	TCAGAAGTTAGCGGATGAGGTA	PCR for identifying RAD51A2 mutation in *Osrad51-1*
Cr-RAD51A2-seq-R	TGCTGTATCCTGAAGGCGATT	PCR for identifying RAD51A2 mutation in *Osrad51-1*
Cr-DMC1A-seq-F	AGTTGTTTGGTGTGATCCTGAC	PCR for identifying DMC1A mutation
Cr-DMC1A-seq-R	TGTTATGAAGCCCTGACTCTGA	PCR for identifying DMC1A mutation
Cr-DMC1B-seq-F	CTCCCTGGGTTTCATTCTTCAT	PCR for identifying DMC1B mutation
Cr-DMC1B-seq-R	ACGCAGATCAGCTCCTATTAAC	PCR for identifying DMC1B mutation
BD-RAD51A1-F	CTGATCTCAGAGGAGGACCTGATG TCGACGTCGGCGGCGGCG	Generate pGBKT7 construct for RAD51A1
BD-RAD51A1-R	TGCAGGTCGACGGATCCCCGGTCA ATCCTTGACATCTGCAAC	Generate pGBKT7 construct for RAD51A1
BD-RAD51A2-F	CTGATCTCAGAGGAGGACCTGATG TCGTCGTCGGGTGCGGCT	Generate pGBKT7 construct for RAD51A2
BD-RAD51A2-R	TGCAGGTCGACGGATCCCCGGTCA GTCCTTAACATCTGTGAC	Generate pGBKT7 construct for RAD51A2
BD-DMC1A-F	CTGATCTCAGAGGAGGACCTGATG GCGCCGTCCAAGCAGTAC	Generate pGBKT7 construct for DMC1A
BD-DMC1A-R	TGCAGGTCGACGGATCCCCGGTCA GTCTTTCGCATCCATTAT	Generate pGBKT7 construct for DMC1A
BD-DMC1B-F	CTGATCTCAGAGGAGGACCTGATG GCGCCGTCCAAGCAGTAC	Generate pGBKT7 construct for DMC1B
BD-DMC1B-R	TGCAGGTCGACGGATCCCCGGTCA GTCTTTCGCATCCATTAT	Generate pGBKT7 construct for DMC1B
AD-BRCA2-F	TACGACGTACCAGATTACGCTATGC AGAGGAGGTGGCAGGTG	Generate pGADT7 construct for BRCA2
AD-BRCA2-R	GTATCGATGCCCACCCGGGTGTCA GCCACCACTGTCACCAAC	Generate pGADT7 construct for BRCA2
AD-FIGL1-F	TACGACGTACCAGATTACGCTATGG CGGAGCAGTCTCACGCC	Generate pGADT7 construct for FIGL1
AD-FIGL1-R	GTATCGATGCCCACCCGGGTGTTAA TTTGCTAAGCTCCCAAA	Generate pGADT7 construct for FIGL1
SCC-RAD51A1-F	GGGCCCAGGCCTACTAGTATGTCG ACGTCGGCGGCG	Generate pSCYCE(R) construct for RAD51A1
SCC-RAD51A1-R	CTACCCGGGAGCGGTACCATCCTT GACATCTGCAAC	Generate pSCYCE(R) construct for RAD51A1
SCC-RAD51A2-F	GGGCCCAGGCCTACTAGTATGTCG TCGTCGGGTGCG	Generate pSCYCE(R) construct for RAD51A2

SCC-RAD51A2-R	CTACCCGGGAGCGGTACCGTCCTT AACATCTGTGAC	Generate pSCYCE(R) construct for RAD51A2
SCC-DMC1A-F	GGGCCCAGGCCTACTAGTATGGCG CCGTCCAAGCAG	Generate pSCYCE(R) construct for DMC1A
SCC-DMC1A-R	CTACCCGGGAGCGGTACCGTCTTT CGCATCCATTAT	Generate pSCYCE(R) construct for DMC1A
SCC-DMC1B-F	GGGCCCAGGCCTACTAGTATGGCG CCGTCCAAGCAG	Generate pSCYCE(R) construct for DMC1B
SCC-DMC1B-R	CTACCCGGGAGCGGTACCGTCTTT CGCATCCATTAT	Generate pSCYCE(R) construct for DMC1B
SCN-FIGL1-F	GGGCCCAGGCCTACTAGTATGGCG GAGCAGTCTCAC	Generate pSCYNE(R) construct for FIGL1
SCN-FIGL1-R	CTACCCGGGAGCGGTACCATTTGC TAAGCTCCCAAA	Generate pSCYNE(R) construct for FIGL1
SCN-BRCA2-F	GGGCCCAGGCCTACTAGTATGCAG AGGAGGTGGCAG	Generate pSCYNE(R) construct for BRCA2
SCN-BRCA2-R	CTACCCGGGAGCGGTACCGCCACC ACTGTCACCAAC	Generate pSCYNE(R) construct for BRCA2
